# Philadelphia Dispensary

**Published:** 1840-07-25

**Authors:** C. N. Berkeley


// MEDICAL EXAMINER.

DEVOTED TO MEDICINE, SURGERY, AND THE COLLATERAL SCIENCES.

No. 30.] PHILADELPHIA, SATURDAY, JULY 25, 1840. [Vol. III.

### PHILADELPHIA DISPENSARY.

*Report for North Middle District, from 1st to 31st July, inclusive.*

| | |
|---|---|
| Whole number of cases | 50 |
| Cured | 40 |
| Relieved | 3 |
| Under treatment (mostly chronic) | 7 |
| Of the cases cured, there were of— | |
| Cholera infantum | 10 |
| of adults | 2 |
| Diarrhœa | 2 |
| Dysentery | 2 |
| Menorrhagia | 3 |
| Fever, remittent | 7 |

The remaining cases generally unimportant, excepting three of cutaneous affections,—one of which, a case of *ecthyma*, is of peculiar interest, owing to its periodical occurrence every summer since that in which the patient, then an infant, was vaccinated.

It will be seen that the prevailing affection of this district, during the month of July, has been Cholera, both of infants and adults. The two cases of adults were of a kind nearly approaching Cholera Asiatica, having the characteristic symptoms of spasms, great pain, and excessive prostration, the surface cold and cadaverous to the touch. The evacuations from the bowels of a thin, serous kind, similar to the rice-water discharge of Asiatic cholera. The fluid thrown from the stomach, differing from that in genuine cholera, apparently pure bile, as if expressed from the liver and gall bladder, by the violent contractions of the abdominal muscles and diaphragm. With this exception, the symptoms of Asiatic cholera prevailed to an alarming degree, the patients exhibiting a disposition to sink within twelve hours after the attack, the surface of the body, and particularly the extremities, being very cold, and the pulse reduced to a mere thread. The violence of the symptoms was relieved by the use of calomel, camphor, and opium; with hot flannels, sprinkled with whisky, applied over the whole surface of the abdomen,—though the system did not react, till brandy was freely exhibited in the form of julep. These cases appear to have been caused by eating cucumbers.

The cases reported as *fever* were mostly complicated with derangement of the stomach and intestines. Though not amounting to actual cholera or dysentery, they were still so marked as to exhibit a strong tendency to assume the type of the prevailing affection of the district.

The three cases of *menorrhagia* occurred nearly at the same time, and within a short distance of each other,—a coincidence a little remarkable in a complaint, the existence of which, as *menorrhagia*, has been denied by some, who have considered it rather a uterine hæmorrhage, than an increased menstrual flow. In these cases there was nothing to justify such a conclusion, and the discharge appears to have been nothing more than a profuse "menstrual secretion."

C. N. Berkeley.

